# Systemic management of malignant meningiomas: A comparative survival and molecular marker analysis between Octreotide in combination with Everolimus and Sunitinib

**DOI:** 10.1371/journal.pone.0217340

**Published:** 2019-06-20

**Authors:** Andrés F. Cardona, Alejandro Ruiz-Patiño, Zyanya Lucia Zatarain-Barrón, Fernando Hakim, Enrique Jiménez, Juan Armando Mejía, Juan Fernando Ramón, Nicolás Useche, Sonia Bermúdez, Diego Pineda, Hernando Cifuentes, Leonardo Rojas, Luisa Ricaurte, Luis Eduardo Pino, Carmen Balaña, Oscar Arrieta

**Affiliations:** 1 Brain Tumor Section, Clinical and Translational Oncology Group, Clínica del Country, Bogotá, Colombia; 2 Foundation for Clinical and Applied Cancer Research–FICMAC, Bogotá, Colombia; 3 Molecular Oncology and Biology Systems Group (G-FOX), Universidad El Bosque, Bogotá, Colombia; 4 Laboratory of Experimental Oncology, Instituto Nacional de Cancerología (INCaN), México City, México; 5 Neurosurgery Department, Fundación Santa Fe de Bogotá, Bogotá, Colombia; 6 Neuroscience Department, Universidad El Bosque, Bogotá, Colombia; 7 Neuroradiology Section, Radiology Department, Fundación Santa Fe de Bogotá, Bogotá, Colombia; 8 Neuroradiology Section, Clínica del Country, Bogotá, Colombia; 9 Neurosurgery Department, Clínica del Country, Bogotá, Colombia; 10 Clinical Oncology Department, Clínica Colsanitas, Bogotá, Colombia; 11 Clinical Oncology Group, Fundación Santa Fe de Bogotá, Bogotá, Colombia; 12 Neuro-Oncology Section, Oncology Department, Hospital Germans Trias I Pujol, Catalan Institute of Oncology–ICO, Barcelona, Spain; Seoul National University College of Pharmacy, REPUBLIC OF KOREA

## Abstract

**Purpose:**

To compare the effectiveness of octreotide/everolimus *vs*. sunitinib for the systemic treatment of recurrent aggressive meningiomas.

**Methods:**

31 patients with recurrent or refractory WHO II or WHO III meningiomas were examined in two reference centers in Colombia. Patients who had systemic treatment (sunitinib, everolimus/octreotide) and a complete follow-up were included. Overall survival (OS), progression-free survival (PFS) and toxicities were evaluated. Additionally, tissue samples were examined for PDGFRβ and VEGFR2, their expression was correlated with outcomes.

**Results:**

Twenty-two patients (72%) were female with a median age of 55 years (SD±15.3). The most prevalent histology was anaplastic meningioma in 20 patients (65%) with 48% of patients suffering from three previous relapses before the start of systemic treatment. A total of 14 patients received combination therapy with octreotide/everolimus, 11 received sunitinib and the remaining 6 received other second-line agents. Median OS was 37.3 months (95%CI 28.5–42.1) and the PFS during the treatment with everolimus/octreotide (EO) and sunitinib (Su) was 12.1 months (95%CI 9.2–21.1) and 9.1 months (95%CI 6.8–16.8); *p* = 0.43), respectively. The OS of the group treated with the EO→Su→Bev sequence (1^*st*^/2^*nd*^*/3*^*rd*^ line) was 6.5 months longer than the Su→EO→Bev sequence (36.0 vs. 29.5 months) (p = 0.0001). When analyzing molecular markers, the positive PDGFRβ and negative VEGFR2 expression were associated with longer survival both in OS and PFS.

**Conclusion:**

Sunitinib and octreotide/everolimus have similar efficacy and safety in the systemic management of refractory meningioma. VEGFR2 and PDGFRβ expression are associated with better outcomes.

## Introduction

Meningiomas are the most common type of intracranial tumor, with an estimated prevalence of around 97.5 cases for each 100.000 individuals in the United States [1, 2]. Tumor grading is performed according to the World Health Organization (WHO) classification for tumors of the central nervous system (CNS), which has been continuously revised in 2000, 2007 and 2016 [3, 4]. Almost all cases (95%) are considered benign (WHO I), whereas the remaining are cataloged as either atypical (WHO II) or anaplastic meningiomas (WHO III) [1, 4]. Grading is strongly correlated with clinical outcomes; tumors catalogued as WHO grade II and III carry a 5 to 10-fold increase in recurrence with 59% and 28% of patients free of relapse at 5 years, respectively [5]. Other endpoints such as median overall survival (OS) and quality of life (QoL) are also strongly affected [6, 7]. Interestingly, higher-grade meningiomas, particularly WHO II, have been increasingly diagnosed in the last years, likely due to improvements in classification criteria stemming from a better understanding of tumor biology [3, 5].

Therapeutic schemes for meningioma patients are usually based on surgical resection and/or radiotherapy. Complete surgical resection is usually the standard, in order to decrease likelihood of recurrence. However, a complete resection might not be possible in cases in which the tumor is located in an anatomically challenging position. For such patients, radiotherapy might be used alone, or following subtotal surgical resection. However, few prospective studies have been conducted to assess the patient and treatment characteristics which will render this intervention most beneficial [5]. Additionally, patients who are treated with radiotherapy alone forfeit the possibility of obtaining an adequate histopathological diagnosis and therefore risk of recurrence cannot be confidently assessed [5].

Despite significant advances in surgical and RT techniques, many patients with meningioma will recur. Recurrence depends on a number of factors, including surgical resection (ranging from 9% to 44% depending on the Simpson grade of resection), radiotherapy series (ranging from 7% to 23% 5-year recurrence rates) and other important variables [5]. Patients with a recurrence will likely face further surgical resections, however this option is limited to those with tumors which can be surgically removed. For patients in whom surgery and radiotherapy are no longer a viable option, the current recommendation is to initiate systemic therapy, however systemic therapy is still lacking robust evidence in order to support the use of specific agents, as well as questions involving lines of treatment, treatment sequencing, targeted agents and assessment of clinical and radiological outcomes [8].

Though a number of systemic therapies are available for treating recurrent meningioma, including chemotherapy, hormonal therapy, targeted therapies and biologic agents, most evidence for their use comes from small phase II studies, with a small number of patients, largely limiting the interpretation of the results [9, 10]. Efforts continue to seek alternatives which can offer patients consistent survival benefits, including an ongoing randomized clinical trial by the EORTC Brain Tumor Group, which is evaluating the use of Trabectedin for recurrent meningioma; these results will likely impact future recommendations (NCT02234050).

Another important limitation for the use of systemic agents is the lack of validated biomarkers in order to select patients who will likely respond to specific agents.

Taking into account previous considerations, no agent or therapeutic sequence has been shown to be largely effective over another. The rationale for this study was to report comparative systemic treatment benefits in progressive atypical or anaplastic meningioma.

## Materials and methods

### Patients and study design

A retrospective review of records of patients with histopathologically confirmed grade II or III meningioma (n = 52) treated at two reference centers of Bogotá, Colombia (Clínica del Country and Fundación Santa Fe de Bogotá) between December 2011 and January 2016 was conducted. During this time period, 31 patients (60%) with recurrent/progressive aggressive meningioma were treated with octreotide acetate LAR [O]/everolimus [E] (30 mg IM q28 days/10 mg PO q/day), sunitinib [Su] (50 mg PO q/day for days 1–28 of 42 days) or bevacizumab [Bev] (10 mg/kg IV days 1 and 15). All patients (n = 15/48.3%) exposed to the somatostatin analogue had over expression of SSTR2 (somatostatin receptor 2). The medical records were reviewed for demographic (age at diagnosis, number of recurrences) and prior treatment characteristics (extent of resection, type and dose of RT), adverse events and outcome. All procedures performed in this study involving human participants were in accordance with the ethical standards of the institutional and national research committee and with the 1964 Helsinki declaration and its later amendments or comparable ethical standards. Informed consent was obtained from all individual participants included in this study. The study was approved by the local ethics/research committees (Clínica del Country (Bogotá, Colombia) ethics/research committees [LR23-2016]).

All patients were over 18 years of age and had radiologic evidence of either progressive or recurrent tumor after prior therapy (surgical resection and/or radiation therapy). Treatment consisted of two different sequences E→Su→Bev (1^*st*^/2^*nd*^*/3*^*rd*^ line) and Su→ E→Bev, according to the choice of the treating group. Every therapy was discontinued after disease progression or toxicity. Patients were evaluated by physical examination every 3 weeks and MRI scans every 8 weeks. Assessment of response was based on Radiologic Assessment in Neuro-Oncology (RANO) criteria that included evaluation of both enhancing and non-enhancing imaging findings as well as clinical changes [5, 11] Routine laboratory studies were assessed each month or sooner if medically indicated. Toxicities were graded according to the National Cancer Institute’s Common Terminology Criteria for Adverse Events, version 4.0.

### Determination of SSTR2A, VEGRF_2_ and PDGFRβ expression

Four-micron sections that contained human meningiomas were cut from formalin-fixed paraffin-embedded tissue blocks. The slides were deparaffinised in a series of histolene (Engelbrecht, Edermuende, Germany) and alcohol solutions. The Vectastain Elite (Vector Laboratories, Burlingame, CA, USA) was used according to manufacturer’s protocol. Briefly, the slides were treated with an antigen unmasking solution (citrate buffer, pH 6.0; 94°C; 30 min), rinsed with PBS, and bathed in 3% H2O2 for 10 min, followed by blocking with avidin/biotin (Abcam ab3387, Abcam, Cambridge, MA, USA). The slides were incubated overnight at 4°C with a Flk-1 (VEGFR_2_) rabbit polyclonal antibody (Ab2349; Abcam, Cambridge, MA, USA) at 1:100 and a PDGFRβ rabbit polyclonal antibody (sc-339; Santa Cruz) at 1:200. Next, the slides were washed three times with PBS, a biotinylated secondary antibody was applied, and a premixed avidin-biotin-peroxide complex was added (ABC; Vector Laboratories). The stains were developed with Vector Novared (Vector Laboratories). The positive controls were selected for each specimen as follows: VEGFR_2_ (human kidney) and PDGFRβ (human placenta). The negative control slides were incubated either without primary antibody or with a blocking peptide. The sections were counterstained with haematoxylin and examined using light microscopy. All immunostained slides were reviewed by an experienced pathologist to render an assessment of VEGFR_2_ and PDGFRβ expression. The level of expression was graded from 0% to 100% based on the percentage of cells that were positive, relative to the total tissue. The expression of VEGFR2 was dichotomized according to the percentage of expression as positive when it was greater than 10%, and negative in those with levels between 0–9%. For the expression of PDGFRβ, it was considered as positive when it was greater than 1%.

Additional cuts were prepared for SSTR2A expression; heat mediated antigen retrieval was performed (with low pH) in automated water bath (Dako PT Link), and sections were stained with the recombinant monoclonal antibody UMB1 (ab134152, anti-Somatostatin Receptor 2 antibody, SSTR2A, Abcam, rabbit monoclonal, 1:100). Staining was performed in an autostainer (Dako autostainer link 48) using a polymer-based detection system (Dako EnVision FLEXTMK8000). Immunohistochemical staining for SSTR2A was scored according to the percentage of immunostained tumour cells (0: less than 5%, 1+: 5% to 25%, 2+: 26% to 50%, 3+: more than 50%). The intensity of staining was then classified as weak, moderate and strong. All intensities were relative to control staining in human pancreas islet cells and were considered positive when the level of expression was equal to or greater than 2+.

Additionally, the KI_67_/MIB_1_ expression was estimated following standardized methods [12] as well as the invasion of bone and adjacent brain tissue.

### Statistical methods

Continuous variables were reported as arithmetic means or medians and ranges. Categorical variables were summarized as proportions with corresponding 95% confidence intervals (95% CI). Survival curves were constructed from time to event variables using the Kaplan-Meier methodology. Statistically significant differences were estimated with the Log-Rank Test. Statistical significance was considered as p≤0.05 using a two-sided test. Statistical analyses were performed using SPSS version 23.0 (IBM Corp. IBM SPSS Statistics for Windows, Armonk, NY: IBM Corp Released 2015) and R version 3.3.3 (The R Foundation, Vienna, Austria).

## Results

### Patient and tumor characteristics

A total of 31 patients with recurrent anaplastic or atypical meningioma were included in the study. Patient characteristics are summarized in **Table 1**. The majority of the population was female in an almost 2:1 ratio with a predominance of diagnosis in patients younger than 60 years old. In terms of location all atypical lesions arose from the convexity (n = 4), the middle (n = 5) or the anterior fossa (n = 2), compared to anaplastic lesions that were located parasagitally (n = 8) or at the anterior (n = 11) or posterior fossa (n = 1). Additionally, a total of 8 patients (26%) suffered from multicentric disease, 6 of which had anaplastic phenotype. Bone involvement was similar for both histologies and was present in nearly half of patients whereas brain invasion occurred in 13 of the cases with grade III tumors.

**Table 1:** Patient characteristics.

Variable	Value	Range or 95%confidence interval
Median age	55 years	28–88 years
Female N (%)	22 (71%)	95CI (55–87%)
**Pathology**	**N (%)**	**95%CI**
WHO II N (%)	11 (35%)	19–52%
WHO III N (%)	20 (65%)	48–81%
**N. of prior recurrences before medical treatment**	**N (%)**	**95%CI**
1	4 (13%)	11–25%
2	9 (29%)	13–45%
3	10 (32%)	16–49%
4	5 (16%)	3–29%
5	3 (10%)	0–20%
**Primary location**	**N (%)**	**95%CI**
Convexity	4 (13%)	11–25%
Parasagittal	8 (26%)	10–41%
Anterior fossa	13 (42%)	25–59%
Middle fossa	5 (16%)	3–29%
Posterior fossa	1 (3%)	0–9%
**Multicentricity**	**8 (26%)**	**10–41%**
**N. of prior Surgeries**	**N (%)**	**95%CI**
1	2 (6%)	0–15%
2	19 (61%)	44–78%
3	6 (19%)	5–33%
4	1 (3%)	0–9%
5	3 (10%)	0–20%
**Extent of resection**	**N (%)**	**95%CI**
Total resection	14 (45%)	27–62%
Subtotal resection	17 (55%)	37–72%

### Treatment

All patients underwent surgical resection, and the median number of partial or radical procedures was 2 (range, 1–5). In the group of patients with atypical tumors (n = 11) all but one (90.9%) had Simpson III resections compared with the group of subjects with anaplastic tumors, among whom 65% (n = 13) had Simpson I and II procedures (p = 0.0089). No differences were found between the two groups according to the number of recurrences (*p* = 0.36). Additionally, all patients were treated either with IMRT (n = 20, 65%) or stereotactic radiosurgery (n = 11, 35%), with a median time elapsed between RT and the beginning of the first line of systemic treatment of 22.9 months (95%CI 1.8–189.0). No differences were found in progression-free survival (PFS) among patients treated with IMRT or stereotactic radiosurgery (23.6 *vs*. 19.4 months; *p* = 0.47). At the time of starting medical therapy, mean tumor diameter was 42.3 mm (SD+/-12.2) with no differences between the two histologies (p = 0.85). Before receiving sunitinib or everolimus-based treatment one subject (3.2%) received hydroxyurea/imatinib without benefit. Nineteen patients received everolimus or everolimus/octreotide as the first line (61.3%) and 11 sunitinib (35.5%). The relationship for the second line was reversed, 18 patients received sunitinib (58.1%), 4 (12.9%) everolimus based treatment, and one bevacizumab (3.2%). In 8 (25.8%) cases, first line treatment is still in progress. After a median follow-up of 31.8 months (95% CI 13.6–47), 10 patients (32.3%) received the sequence E→Su→Bev, 9 patients (29.0%) received E→Su, and 4 (12.9%) were exposed to the sequence Su→E→Bev.

### Treatment outcomes

**Fig 1:**
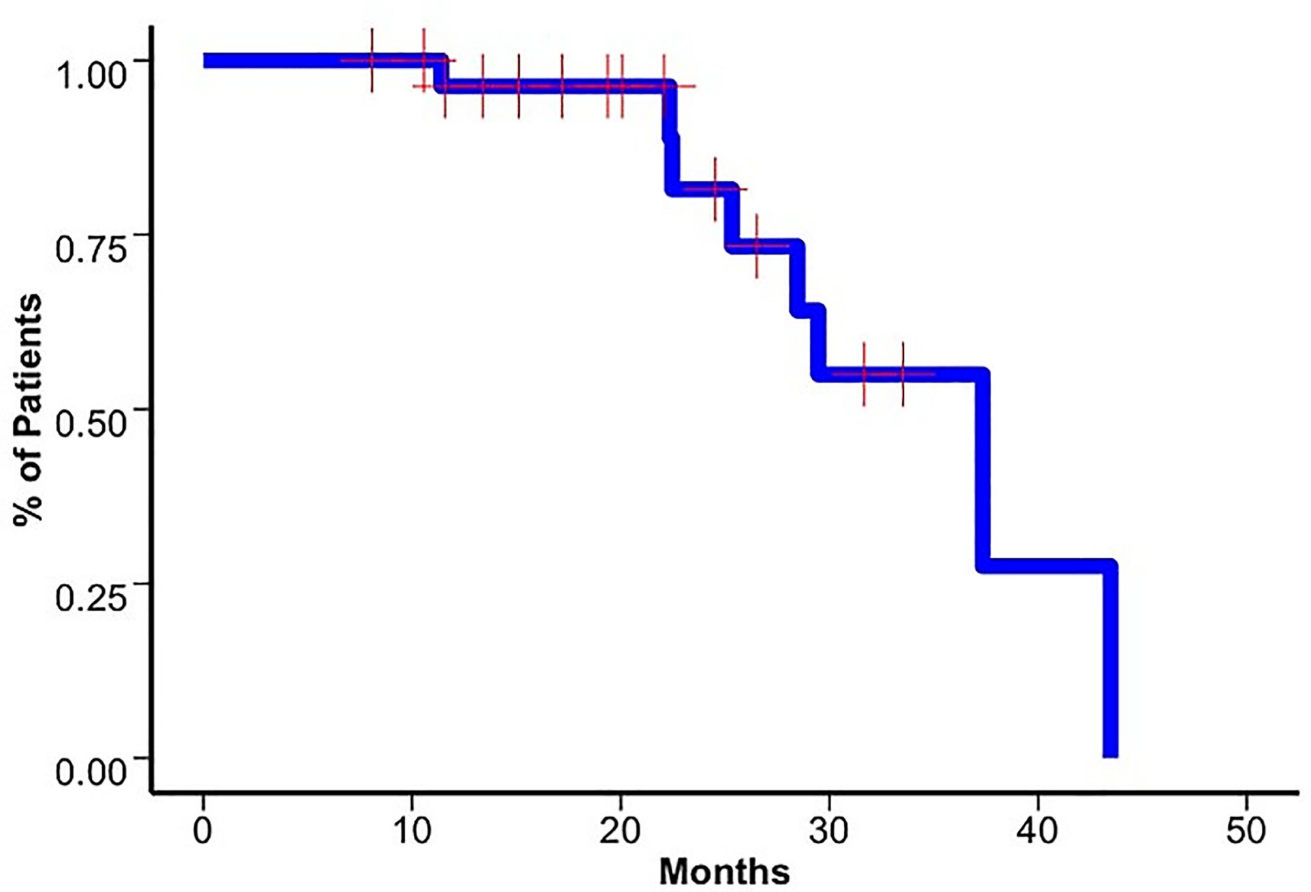
Overall survival for entire cohort. Kaplan–Meier curve for overall survival (OS) for the entire study cohort after medical treatment initiation (37.3 months, 95%CI 28.5–42.1).

**Fig 2:**
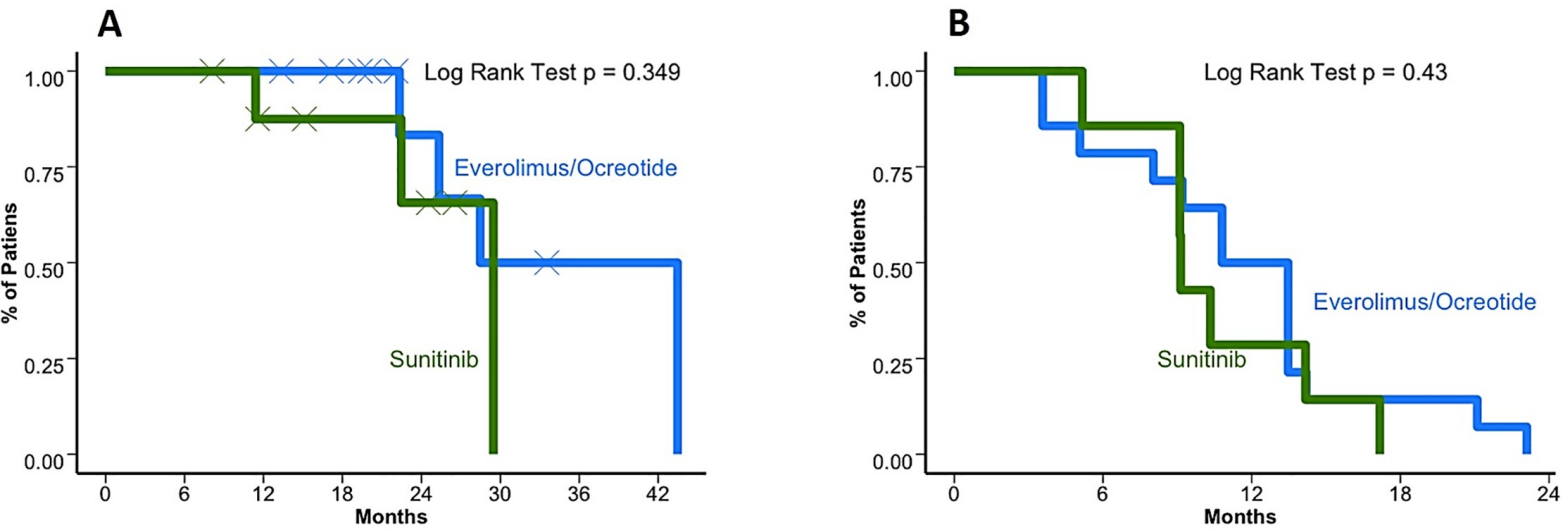
Overall survival and progression free survival for first-line Everolimus+octreotide and sunitinib (A) Kaplan–Meier curve for first line overall survival (OS). Everolimus±octreotide and sunitinib offered a median of 36 months (95%CI 25.3–41.7) and 29.5 months (95%CI 22.5–37.5; *p* = 0.349), respectively. (B) Kaplan–Meier curve for first line progression free survival (PFS). Everolimus±octreotide and sunitinib offered a median of 12.1 months (95%CI 9.2–21.1) and 9.1 months (95%CI 6.8–16.8; *p* = 0.43), respectively.

**Fig 3:**
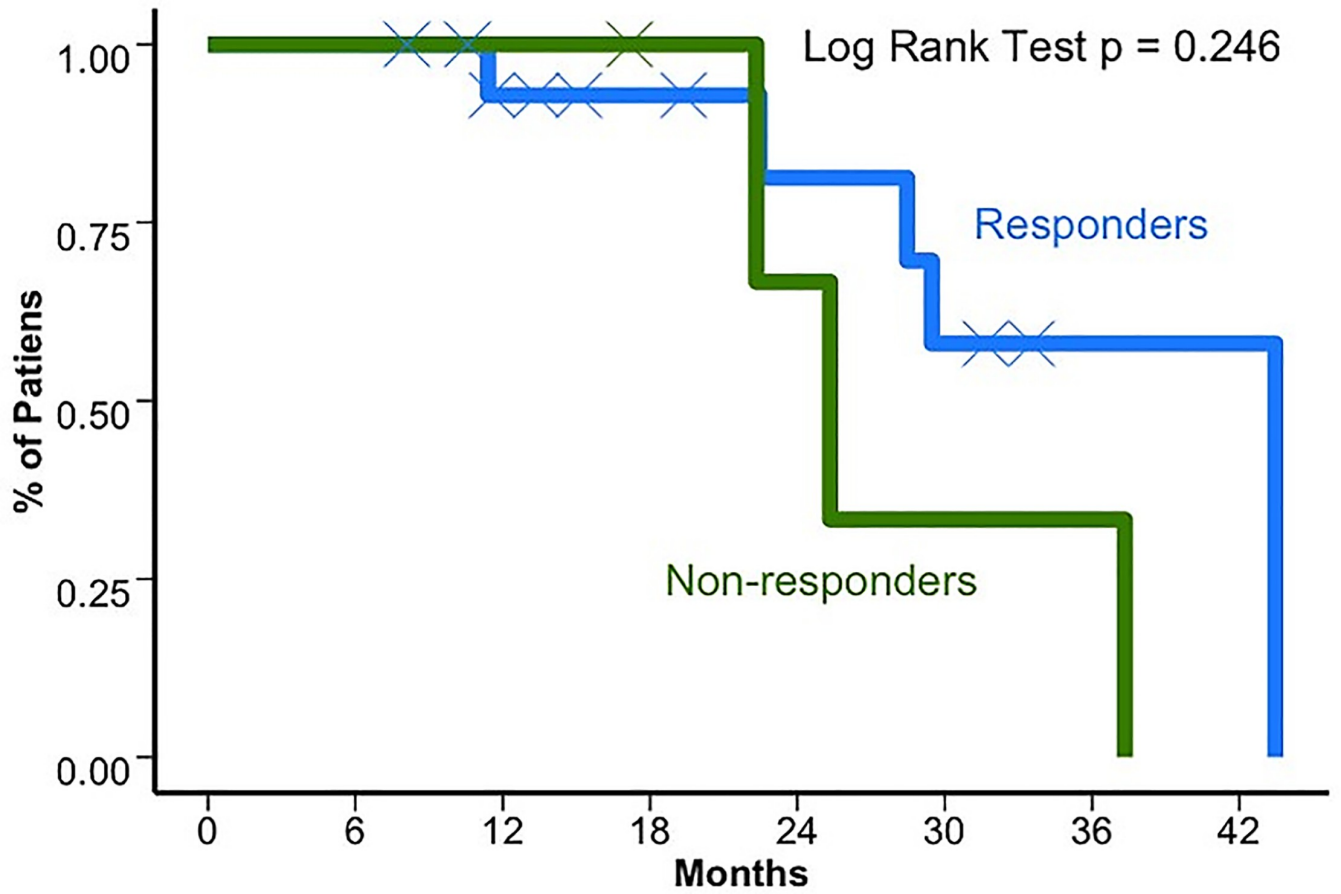
Overall survival based on response to first line therapy. For responders and non-responders OS were 43.5 (95%CI 28.5-NR) and 25.3 (95%CI 22.3-NR) months, respectively.

The median OS for the cohort after medical treatment initiation was 37.3 months (95%CI 28.5–42.1) and 78.1 months (95%CI 42.5–98.2) after initial diagnosis (**Fig 1**). When comparing patients treated in first line with the two most common strategies, everolimus±octreotide or sunitinib the median PFS was 12.1 months (95%CI 9.2–21.1) in comparison to 9.1 months (95%CI 6.8–16.8; *p* = 0.43), respectively. In terms of OS, everolimus±octreotide and sunitinib offered a median of 36 months (95%CI 25.3–41.7) and 29.5 months (95%CI 22.5–37.5; *p* = 0.349), respectively. The OS and PFS for the first line of treatment are presented in **Fig 2A** and **2B**. On the other hand, response rate (RR) for first line according to the RANO criteria were 6 (43%) patients that achieved stable disease (SDis) and 4 (28.6%) who achieved partial response (PR) with everolimus±octreotide in contrast to 7 (63.6%) patients who achieved stable disease and 4 (36.4%) partial response when receiving sunitinib. The clinical benefit (SDis+PR) showed a tendency towards prolonging OS without a definitive statistically significant association (*p* = 0.246) as observed in **Fig 3**. The PFS for patients who received sunitinib and everolimus± octreotide in the second line was 9.13 (95%CI 2.4–13.6) and 10.17 (95%CI 6.13–14.5), respectively. OS was superior when the sequence E→Su→Bev was used versus all other possibilities (*p* = 0.0001). But not by the extension of the initial surgery (*p* = 0.94), by the time between diagnosis and the start of medical treatment (< or >20 months; *p* = 0.17), by the number of recurrences (< or >3 recurrences; *p* = 0.47), by age (< or >65 years, *p* = 0.64) or by gender (*p* = 0.30). None of the clinical variables influenced multivariate analysis for OS. **S1 Fig** shows the clinical and imaging evolution of two patients treated with the E→Su→Bev and Su→E→Bev sequences.

### Pathological characteristics and molecular markers

**Fig 4:**
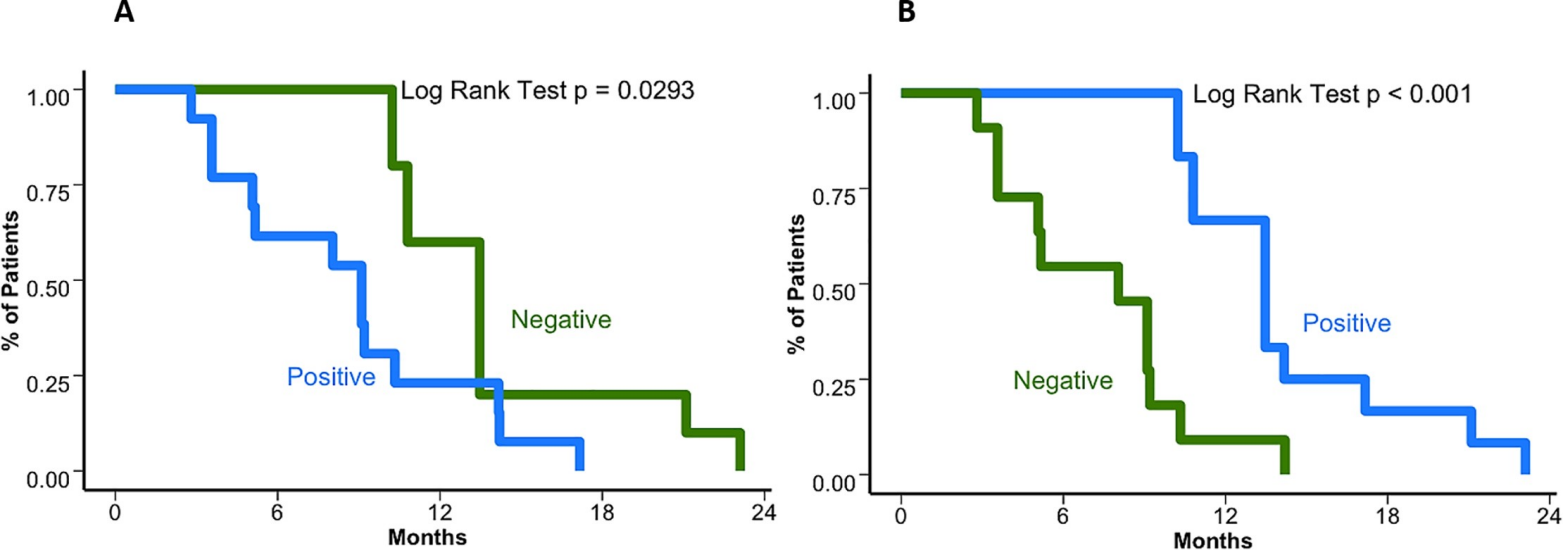
Survival outcomes based on VEGFR2 and PDGFRβ expression. (A). Kaplan–Meier curve for progression free survival (PFS) based on VEGFR2 expression. The median PFS when VEGFR2 expression is positive was 9.1 months (95%CI 5.07–13.4) *vs*.13.5 months when protein expression was negative (95%CI 10.8–17.2) (*p* = 0.029). (B) Kaplan–Meier curve for progression free survival (PFS) based on PDGFRβ expression. The median PFS when there is positive reactivity was 13.5 months (95%CI 10.8–15.4) vs 8.0 (95%CI 5.07–10.2) (*p*<0.001) for those who were negative.

A high expression of VEGFR and PDGFRβ (**S2A and S2B Fig**) was found in 54.8% (n = 17) and 61.3%, respectively. Thirteen patients (42%) had a positive expression of SSTR2A (**S3 Fig**), group that was exposed to octreotide in combination with everolimus. Nineteen cases (61.3%) had Ki_67_ >20% and hypercellularity, and 67% had lesions with prominent nucleoli. VEGFR2 overexpression was associated with a shorter OS (27.4 months [95%CI 22.5–31.7] *vs*. 43.5 months [95%CI 34.8–42.4] for those without immunoreactivity; *p* = 0.019). In terms of median PFS, patients with VEGFR2 overexpression also had a lower time of disease control with first line treatment (9.1 months [95%CI 5.07–13.4] *vs*.13.5 months when protein expression was negative [95%CI 10.8–17.2]; *p* = 0.029) On the other hand, PDGFRβ expression was inversely associated with survival outcome, favorably associated with an improved OS (43.5 months [95%CI 32.1–44.5] vs 25.3 [95%CI 22.1–30.5] months, p = 0.0045) and PFS (13.5 months [95%CI 10.8–15.4] months vs 8.0 [95%CI 5.07–10.2]; *p*<0.001) when the immunophenotype showed a positive reactivity. Survival curves are presented for PFS according to VEGFR2 and PDGFRβ in **Fig 4A** and **4B**, respectively. Multivariate analysis conducted for both the expression of VEGFR2 and PDGFRβ reveal several associations. VEGFR2 expression was only positively associated with extent of resection (*p* = 0.025). On the other hand, PDGFRβ was positively associated with multicentricity (*p* = 0.014) and MIB1 (*p* = 0.01) and negatively with brain invasion (*p* = 0.006). Cox regression revealed that MIB1, presence of small cell histology, brain and bone involvement were associated with a shorter OS (*p*<0.001). No associations with PFS were found.

### Toxicities

All patients treated with main sequences experienced some kind of toxicity according to the CTCAE 4.0. The most common side effects for everolimus±octreotide (independent to the treatment line) included grade 1/2 fatigue in 12 (86%) and grade 1/2 oedema in 2 (14%) of patients. In comparison to those exposed to sunitinib who experienced grade 1/2 fatigue in 7 (73%) and hypothyroidism in 2 (18%). Three patients treated with sunitinib experienced grade 3 fatigue which led to a dose reduction and two patients had deep vein thrombosis when exposed to bevacizumab.

## Discussion

Aggressive meningiomas continue to be a difficult to treat pathology. Due to its low frequency, few systemic treatment options have been proven to be effective. Similar to other tumors, a better understanding of the molecular mechanisms involved in tumor growth and progression has increased the probability of identifying successful treatment targets. To the authors´ knowledge this is the first study evaluating the comparative effectiveness of octreotide and everolimus *vs*. sunitinib, reaching similar results in terms of volumetric response and both clinical effectiveness and safety profiles with similar conclusions to the one arm, phase II studies of each individual treatment modality.

Interestingly, the majority of meningiomas express somatostatin receptors, with SSTR2 being the most common detected in 100% of tumors [13]. Due to its high frequency and potential involvement in tumor growth, two agents, pasireotide and octreotide, targeting the aforementioned pathway were investigated. Although early results in a small scale study indicated a possible benefit for octreotide in maintaining stable disease [14], larger studies for both agents failed to demonstrate a statistically significant difference in terms of progression risk or survival [15, 16].

Merlin, encoded by the NF2 gene, is dysregulated in a large fraction of these tumors. By being implicated in multiple signaling transduction pathways such as ERK and Pi3K-AKT, it is considered a key driver in meningioma tumorigenesis. Another possible effect of the alteration of Merlin inhibition is the increased activity of mTORC1, thus potentiating a parallel signaling cascade. This phenomenon led to the investigation of the efficacy of treatment with mTOR inhibitors which proved to be ineffective due to a feedback activation of the Pi3K pathway, perpetuating cellular growth. In response to these findings, multimodal treatment in form of a somatostatin analogue and an mTOR inhibitor showed interesting results in an in vitro study. Cellular viability of tumor samples was negatively affected by each agent individually but the effect was potentiated as the cells were exposed to the combination [17]. This led to a single arm phase II study investigating the treatment with everolimus in combination with octreotide in 37 patients with recurrent or refractory meningioma. This group achieved a median PFS of approximately 9 months, a tolerable safety profile, and a decrease in tumor growth rate thus leading to the maintenance of stable disease in the majority of patients [18].

Furthermore, the family of VEGF receptors has also been proposed as a potential treatment target. Although the pathophysiological mechanism of its contribution to tumorigenesis is not well understood, some authors suggest that the malignant cells of the tumor stroma secrete VEGF-A protein, leading to increased capillary permeability and peritumoral brain oedema [19]. Possibly, by causing a disruption in the microcapillary circulation, agents such as Bevacizumab and Sunitinib could offer a potential treatment modality. Sunitinib, a multitarget kinase inhibitor with activity against VEGF and PDGF has shown promising results in a phase II, one arm study. In this trial, 36 patients received this medication until disease progression or high grade toxicity; achieving a median PFS of 5.2 months and an overall response rate of 6% with stable disease in 69% [20]. Similar to the results of the combination study with everolimus and octreotide, stable disease was the most relevant result and significant contribution of both studies.

Results from this present study also further validate the importance of VEGFR2 as a prognostic marker. The before-mentioned study of Sunitinib evaluated its expression and found its relevance as a predictor of response. Positive expression correlated with a 6.4 median PFS compared to 1.4 months for negative expression [20]. In the present study, median PFS was longer for both VEGFR2 positive and negative patients (ca. 8 vs 14 months) compared to the published findings, while also maintaining the difference in favor of positive patients. This prolonged survival could be possibly explained by the administration of subsequent treatments. Since both groups were tested for VEGFR2, this marker seems to be independently prognostic of administered medication. On the one hand PDGFRβ expression was strongly negatively correlated with VEGFR2 expression. Only 6 patients (19.4%) had both a positive VEGFR2 and PDGFRβ or a negative expression of both factors. This suggests an inverse relationship between these factors as a possible role in the tumor pathophysiology. On the other hand, Sunitinib with is effectiveness due to both VEGF and PDGF pathway inhibition, could be ideally implemented in this subset of patients.

Limitations of the study due to its non-randomized and retrospective nature are worth mentioning. First, selection of treatment was dictated by the treating oncologist based on several factors including adverse events profile, patient preference based on route of administration (oral, intravenous, etc.), and also importantly, the availability and willingness to pay of the insurers. Since no actual randomization was conducted a clear potential selection bias is present. Analyzing the patients´ characteristics, both groups seemed to be roughly similar. Although these results could suggest that both groups could be comparable, non-controlled variables that where not explored in the study could still present a latent bias. Conducting a randomized controlled study is still recommended. Another potential source of bias is the crossover after progression or intolerability of the first line medication. Although no differences were observed in terms of survival endpoints in this setting, it is worth noting that the benefit was observed when the treatment sequences were compared. Crossover between the groups could be a relevant confounder in terms of OS. If a medication could offer a clear benefit in OS over the other, the fact that the patients will be eventually exposed changes the survival profile in the follow-up. Additionally, other factors to be mentioned are the dynamic number of patients in the sequences, the variable time during which a patient received the medication and finally, the hypothesis that the previous exposure of a medication which inhibits a different pathway could present a synergistic or antagonistic effect. On the other hand, the positive association between PDGFRβ negativity and prolonged OS could potentially be explained by the association between its expression and presence of other factors such as MIB1 and brain invasion. In terms of PFS this was not the case suggesting that PDGFRβ could be a better predictor in this specific outcome.

## Conclusions

In conclusion, the present study did not find any statistically significant differences in terms of survival depending on medication administered as first line treatment. However, our data favor the hypothesis of better performance in terms of OS for the sequence E→Su→Bev. Furthermore, it also validates the prognostic significance of VEGFR and PDGFRβ. These results should be validated in a larger randomized controlled trials in order to overcome limitations of the present study.

## Supporting information

S1 Fig Atypical meningioma. A 52-year-old woman with a history of frontal and parasagittal atypical meningioma (12 years of disease evolution; SSTR2A positive, PDGFRb positive and VEGFR negative expression profile) treated in 4 occasions with optimal surgery, in addition to IMRT, radiosurgery and with the sequence E→Su→Bev (OS from medical treatment 33.5 months). **2A.** A 67-year-old woman with a history of frontal and parasagittal anaplastic meningioma (5.2 years of evolution) treated on 2 occasions with optimal surgery, IMRT and with the sequence Su→E→Bev (OS from medical treatment 26.5 months).
(TIF)

S2 Fig VEGFR2 and PDGFRβ immunohistochemistry. Representative examples of immunohistochemical analysis of paraffin embedded high grade meningioma with positive expression for VEGFR2 **(A)** (rabbit polyclonal antibody—Ab2349, Abcam, Cambridge, MA, USA) at 1:100 dilution and for PDGFRβ **(B)** (rabbit polyclonal antibody—sc-339; Santa Cruz, CA, US) at 1:200 dilution.
(JPG)

S3 Fig SSTR2a Immunohistochemistry. Immunohistochemical analysis of paraffin embedded high grade meningioma tissue labeled with ab134152 (Anti-Somatostatin Receptor 2 antibody [UMB1], SSTR2a) at 1/100 dilution. SSTR2a immunohistochemical stain shows strong, diffuse membranous and cytoplasmic positivity in tumor cells.
(TIF)

S1 File (SAV)
