# Supplementary figures and images for: Systemic management of malignant meningiomas: A comparative survival and molecular marker analysis between Octreotide in combination with Everolimus and Sunitinib
Source: PLoS One. 2019 Jun 20;14(6):e0217340. doi: 10.1371/journal.pone.0217340 (PMC6586269; doi:10.1371/journal.pone.0217340)

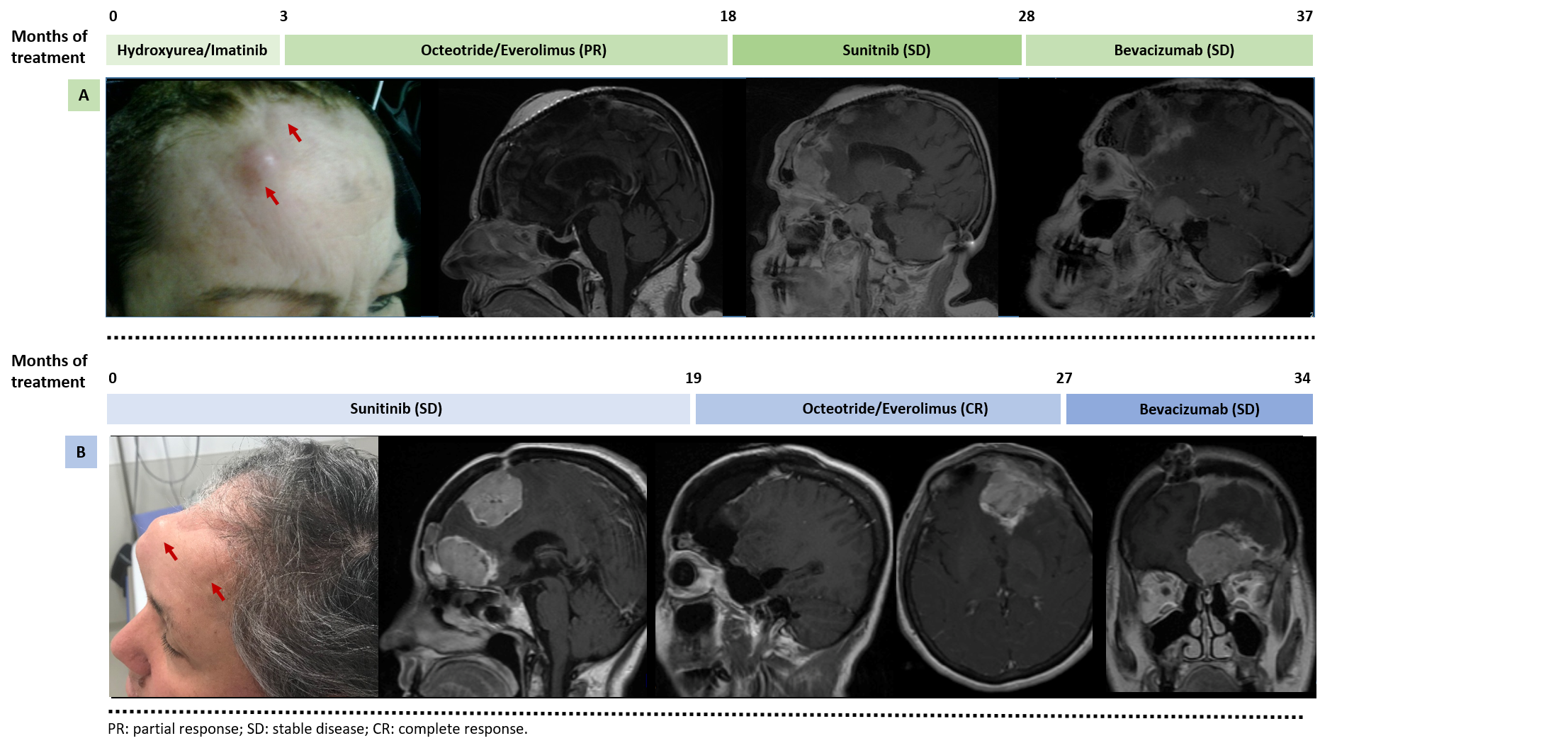

Supplement: S1 Fig — A 52-year-old woman with a history of frontal and parasagittal atypical meningioma (12 years of disease evolution; SSTR2A positive, PDGFRb positive and VEGFR negative expression profile) treated in 4 occasions with optimal surgery, in addition to IMRT, radiosurgery and with the sequence E→Su→Bev (OS from medical treatment 33.5 months). 2A. A 67-year-old woman with a history of frontal and parasagittal anaplastic meningioma (5.2 years of evolution) treated on 2 occasions with optimal surgery, IMRT and with the sequence Su→E→Bev (OS from medical treatment 26.5 months). (TIF) [file pone.0217340.s001.tif]

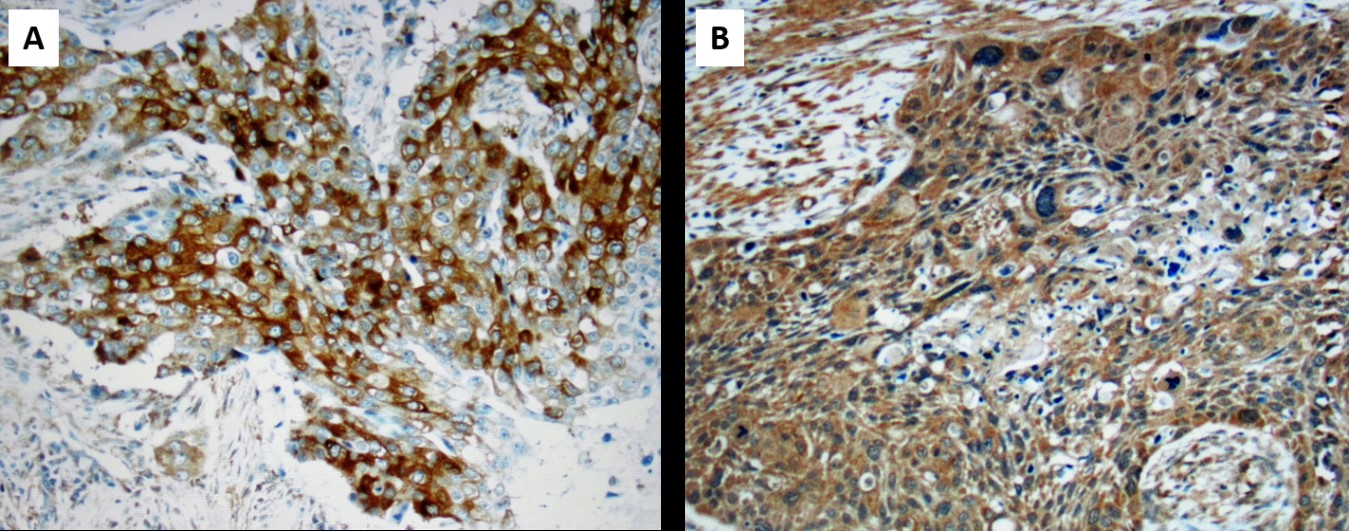

Supplement: S2 Fig — Representative examples of immunohistochemical analysis of paraffin embedded high grade meningioma with positive expression for VEGFR2 (A) (rabbit polyclonal antibody—Ab2349, Abcam, Cambridge, MA, USA) at 1:100 dilution and for PDGFRβ (B) (rabbit polyclonal antibody—sc-339; Santa Cruz, CA, US) at 1:200 dilution. (JPG) [file pone.0217340.s002.jpg]

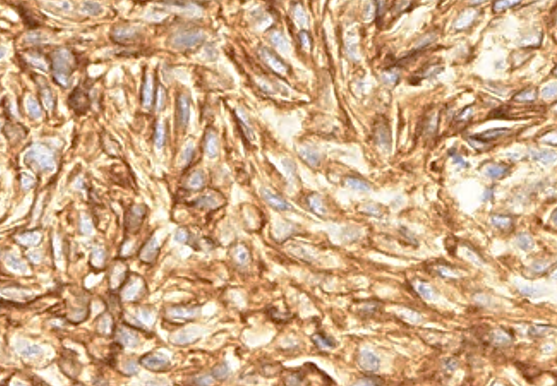

Supplement: S3 Fig — Immunohistochemical analysis of paraffin embedded high grade meningioma tissue labeled with ab134152 (Anti-Somatostatin Receptor 2 antibody [UMB1], SSTR2a) at 1/100 dilution. SSTR2a immunohistochemical stain shows strong, diffuse membranous and cytoplasmic positivity in tumor cells. (TIF) [file pone.0217340.s003.tif]
